# Supplementary material for: New Concept and Apparatus for Cytocentrifugation and Cell Processing for Microscopy Analysis
Source: Int J Mol Sci. 2021 Jul 1;22(13):7098. doi: 10.3390/ijms22137098 (PMC8268716; doi:10.3390/ijms22137098)
Supplement: Supplementary file 1 [file ijms-22-07098-s001.zip › Supplementary Data.pdf]

Supplementary Data

New concept and apparatus for cytocentrifugation and cell processing for microscopy analysis

Anna Ligasová* and Karel Koberna*

Institute of Molecular and Translational Medicine, Faculty of Medicine and Dentistry, Palacký University Olomouc, Hněvotínská 5, 779 00 Olomouc, Czech Republic; anna.ligasova@upol.cz; ka-rel.koberna@upol.cz
* Correspondence: anna.ligasova@upol.cz (AL); karel.koberna@upol.cz (KK)

1. **Supplementary Figures**
2. **Supplementary Videos**

1. Supplementary Figures

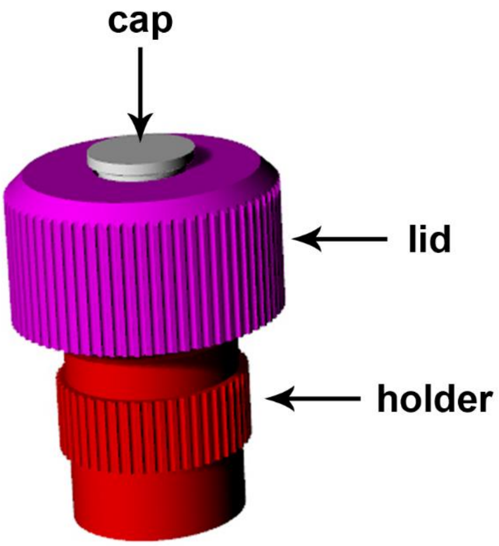

Figure S1. The cap. The CytoTrap can be furnished with the cap closing the opening of the insert during the centrifugation if necessary.

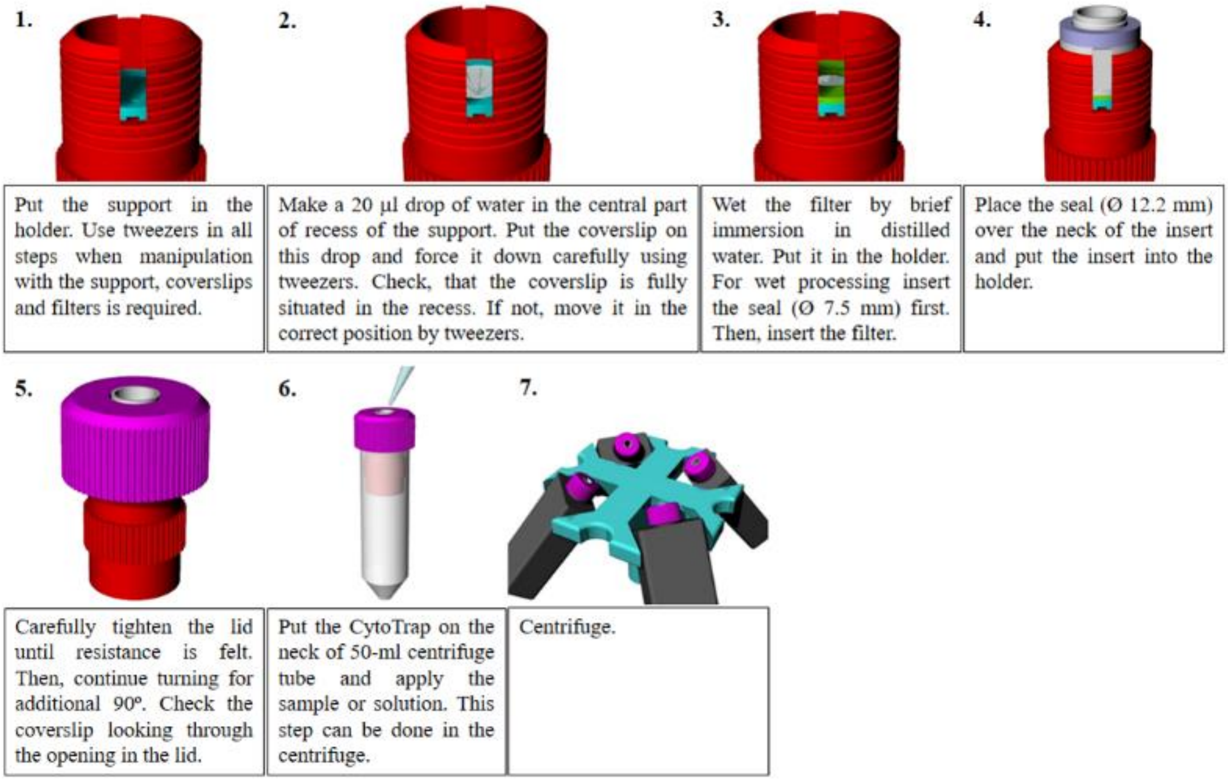

Figure S2. Description of the CytoTrap assembly. The particular steps of the CytoTrap assembly are shown.

2. Supplementary Videos

Video S1. Assembly of the developed device

The step-by-step assembly of the developed device used for the preparation of dry cytospin samples using a common centrifuge with a swing-out rotor.

Video S2. The use of the device in centrifuge

The use of the developed device in a common centrifuge with a swing-out rotor is shown. The application of the samples is performed inside the centrifuge. The 50-ml centrifuge tube is used as a reservoir of the liquid waste.
